# Effect of preemptive analgesia with intravenous oxycodone in the patients undergoing laparoscopic resection of ovarian tumor

**DOI:** 10.12669/pjms.312.6686

**Published:** 2015

**Authors:** Na Wang, Yuantao Wang, Lei Pang, Jinguo Wang

**Affiliations:** 1 Na Wang, Department of Anesthesiology, The First Hospital of Jilin University, Jilin, China; 2 Yuantao Wang, Department of Urology, The First Hospital of Jilin University, Jilin, China; 3 Lei Pang, Department of Anesthesiology, The First Hospital of Jilin University, Jilin, China; 4 Jinguo Wang, Department of Urology, The First Hospital of Jilin University, Jilin, China

**Keywords:** Oxycodone, Preemptive analgesia, Laparoscopy, Pneumoperitoneum, Postoperative pain

## Abstract

**Objective:**

To evaluate the efficacy of preemptive intravenous oxycodone in the patients undergoing laparoscopic resection of ovarian tumor.

**Methods:**

Sixty ASA I or II patients undergoing elective laparoscopic resection of ovarian tumor were randomly allocated to one of two groups: Group O (n=30) received intravenous oxycodone (0.1 mg·kg^-1^) 10 minutes before surgery over 2 minutes, and Group N (n=30) received an equivalent volume of normal saline. All patients received a standardized general anesthesia. MBP and HR at the time of arrival of the operating room (T1), 5 min before pneumoperitoneum (T2), 5 minutes (T3), 10 minutes (T4), and 15 minutes after pneumoperitoneum (T5), and VAS scores at postoperative 2, 4, 8, 12 and 24 hour were recorded. The tramadol consumption and side effects in 24 h after surgery were recorded.

**Results:**

VAS pain scores at 2, 4, 8 and 12 hour after operation were significantly lower in Group O (*P*<0.05). MBP and HR increased significantly due to pneumoperitoneum at T3, T4 and T5, compared with T1 and T2 within Group N, and were higher at T3, T4 and T5 in Group N than at the same time points in Group O. Tramadol consumption was statistically lower in Group O (*P*=0.0003).

**Conclusions:**

Preemptive intravenous oxycodone was an efficient and safe method to reduce intraoperative haemodynamic effect and postoperative pain.

## INTRODUCTION

Laparoscopic surgery has small skin incisions, but intraoperative stress reaction and postoperative pain are still common. Intraoperative stress reaction results from pneumoperitoneum and exploration of pelvic organs. Pain following laparoscopic surgery is multifactorial. Port pain, pelvic organ nociception, diaphragmatic irritation from residual pneumoperitoneum, urinary catheter discomfort contribute to the total pain experience.^1^-^4^ The preemptive concept involves administering an analgesic agent before the onset of the painful stimulus, thus, it may reduce or abolish the development of pain hypersensitization, resulting in less post-stimulus pain. It was shown that preemptive analgesia provides a reduction in perioperative stress, a reduction in postoperative morbidity, an improvement in patient satisfaction. Opioids are frequently used for that purpose.^5^ Oxycodone, a kind of opioids, is superior in the treatment of visceral pain with less side effect than morphine.^6^ Some published studies have revealed that oral oxycodone could decrease intraoperative stress, reduce postoperative opioid consumption and improve pain scores during and after laparoscopic cholecystectomy and liposuction.^7^,^8^ To the best of our knowledge, no comprehensive data exist with regard to the analgesic efficacy of intravenous oxycodone in patients undergoing laparoscopic resection of ovarian tumor.

In this prospective, randomized, placebo-controlled, double-blinded clinical trial, the hypothesis was tested that preemptive intravenous oxycodone would blunt cardiovascular responses to pneumoperitoneum, reduce postoperative pain, and decrease the amount of analgesics used in the postoperative period in patients undergoing laparoscopic resection of ovarian tumor.

## METHODS

Following approval of local ethics committee and written consent from the patients, 60 ASA I-II patients scheduled for laparoscopic resection of ovarian benign tumor were admitted to the study. The patients were double blindly randomized into two groups (Group O n=30, Group N n=30). Simple randomization was accomplished with a computer-generated sequence of numbers and sealed envelopes were used to allocate patients into two groups. Patients with histories of substance abuse and mental illness, who have allergic reactions to study drugs, with liver or renal dysfunctions, pregnant women and ASA III and above were excluded. Routine monitorization (ECG, non-invasive BP and SpO_2_) was performed to patients taken to the operation room without premedication. A 16 gauge intravenous canula was sited. Lactated Ringer’s solution was given at the rate of 8~10 ml·kg^-1^·h^-1^. Anesthesia induction was performed with 0.3 mg·kg^-1^ etomidate, 3 µg·kg^-1^ fentanyl and 0.15 mg·kg^-1^ cisatracurium. Anesthesia was maintained with continuous infusions of propofol and remifentanil at the rates of 6 to 8 mg·kg^-1^·h^-1^ and 0.012 mg·kg^-1^·h^-1^ respectively, and cisatracurium was administrated intermittently as needed. The oxycodone group (Group O) received 0.1mg·kg^-1^ oxycodone (oxycodone hydrochloride injection, HAMOL LIMITED, Thane Road, Nottingham, NottingHamshire, NG90 2DB, U.K.) diluted with normal saline to obtain a concentration of 1 mg·ml^–1^ intravenously over 2 min 10 min before skin incision. The normal saline group (Group N) received 0.1 ml·kg^-1^ saline intravenously over two minutes 10 minutes before skin incision. Following surgery, 100 mg tramadol was administered intravenously if the VAS score was more than 4.

MBP and HR at the time of arrival at the operating room (T1), 5 minutes before skin incision (T2), 5 minutes (T3), 10 minutes (T4) and 15 minutes after pneumoperitoneum (T5) were recorded. The visual analogue scale (VAS: 0- no pain; 1, 2, 3- mild pain; 4, 5, 6- moderate pain; 7, 8, 9- severe pain; 10- the worst pain the patient had ever experienced) scores were recorded at 2, 4, 6, 12 and 24 h after the end of surgery. The total amount of analgesic consumption in the first 24 h postoperatively was recorded. The incidence of postoperative adverse effects (such as nausea, vomiting, itching and dizziness) in the first 24 hour was evaluated with a “yes” or “no” survey. Nausea was defined as a subjective unpleasant sensation associated with the awareness of the urge to vomit. Vomiting was defined as the forceful expulsion of liquid gastric contents.

The primary endpoint of the study was to evaluate 30% or more decrease in tramadol consumption among the groups at estimated time intervals postoperatively. Power analysis with two-sided α of 5% and β of 20% showed that 20 subjects were required in each group. We decided to enroll 30 patients per group for possible dropouts.

Statistical analysis was performed using SPSS 17. The results were shown as mean±standard deviation (SD) and 95% confidence intervals (CIs) of differences were calculated. One Way Variance Analysis (One-Way ANOVA) was used for demographic data of patients, surgical characteristics and tramadol consumption between two groups. MBP and HR were evaluated with One-Way ANOVA between groups; and with Variance Analysis with Repeated Measurements (ANOVA with repeated measurements) within the same group between five different time points. The Mann–Whitney U test was used for VAS scores. We used Fisher exact or Chi Square tests to compare the variables of adverse effects between two groups. A *P*<0.05 was considered significant.

## RESULTS

There was no significant difference between two groups based on demographic data and surgical characteristics (*P*>0.05) (Table-I). VAS scores at 2, 4, 8 and 12 hour after operation were significantly lower in Group O (Table-II) (*P*<0.05).

**Table-I:** Demographic data and surgical characteristics (n=60).

	n	Age (yr)	Weight (kg)	Duration of surgery (min)	Duration of anesthesia (min)
Group O	30	29.2±10.6	54.4±5.2	63.9±15.2	94.7±24.1
Group N	30	30.7±11.4	53.3±4.4	61.1±17.4	97.9±22.2
P		0.59	0.38	0.51	0.59

**Table-II:** VAS scores at various time points postoperatively.

	2h	4h	8h	12h	24h
Group O	1.5±1.4	1.2±1.1	1.5±1.2	1.8±1.0	2.2±1.1
Group N	2.3±1.3	2.1±1.3	2.4±0.9	2.4±0.8	2.5±1.4
P	0.043	0.013	0.001	0.019	0.357

**Fig.1:**
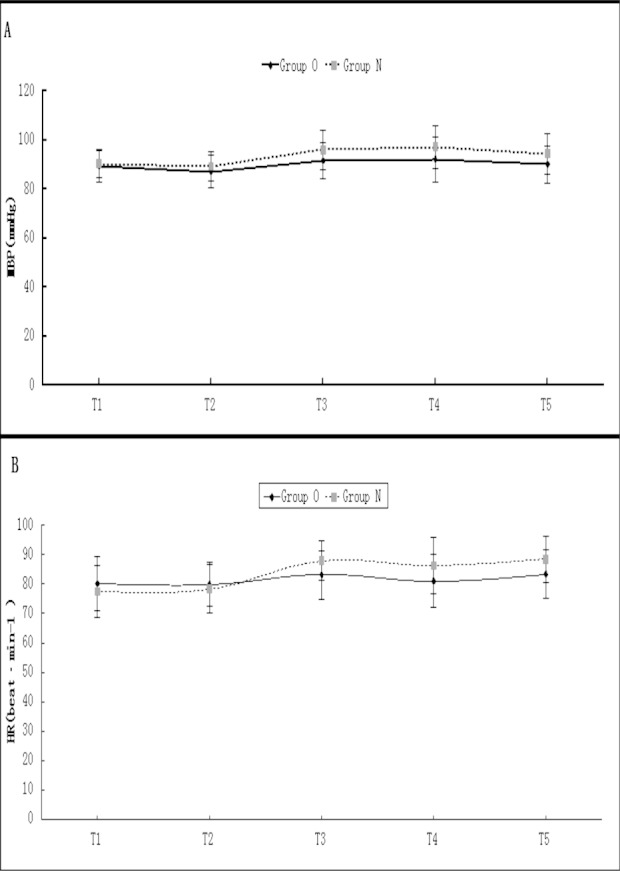
Hemodynamic data for each time point.

Hemodynamic values at each time point were presented in Fig-1. MBP and HR increased due to pneumoperitoneum in the two groups, and significantly increased at T3, T4 and T5 in Group N, compared with T1 and T2 within the same group. MBP and HR were higher at T3, T4 and T5 in Group N than at the same time points in Group O. (Fig-1). The incidences of nausea, vomiting, dizziness and itching were comparable between two groups during the 24 hour observation period (*P*>0.05). Tramadol consumption was statistically lower in Group O (*P=*0.0003) (Table-III).

**Table-III:** Side effects and tramadol consumption in 24 h postoperatively (n, mg).

	Nausea	Vomiting	Dizziness	Itching	Tramadol consumption(mg)
Group O	5	0	4	1	123.6±74.5
Group N	3	1	6	2	204.2±86.2
P	0.70	1.0	0.73	1.0	0.0003

## DISCUSSION

Analgesic intervention before the surgical stimulus, that is, preemptive analgesia may attenuate or block sensitization and hence reduce acute pain.^4^ Although many drugs have demonstrated the evidence of preemptive analgesic benefit, treatments that are likely to prevent the development of central excitability may have the greatest benefit.^4^,^9^ Oxycodone, a kind of opioids, is an interesting option for this purpose. One hour after intravenous administration, oxycodone concentration in plasma was three times as high as that in spinal fluid. At the same plasma concentration, oxycodone had more powerful effect than morphine, which indicated that oxycodone has strong analgesic effect on central nerve system, because of higher concentration in brain.^10^,^11^,^12^ However, the mechanism of the analgesic action of oxycodone is still poorly defined.

Our study demonstrated that intravenous oxycodone administered 10 minutes before surgical incision has preemptive effect on intraoperative hemodynamic changes, reduced postoperative pain and tramadol requirements during the first 24 hour after laparoscopic resection of ovarian tumor. We recorded VAS at rest to assess postoperative pain intensity, because it is easy for the patient to use.

The hemodynamic parameters were studied as an indirect reflection of intraoperative stress reaction in two groups. Patients in Group N had higher BP and HR after pneumoperitoneum and during exploration of pelvic organs. This implied that patients in Group N responded more strongly to surgical stimulation. The low mean BP and HR in Group O at 5, 10 and 15 minutes after pneumoperitoneum corresponded well with the preemptive analgesic effect of oxycodone as compared with Group N.

The role of preemptive analgesia with controlled-release oxycodone one hour before surgery has been previously reported in laparoscopic cholecystectomy. Preemptive controlled-release oxycodone group showed lower pain scores and rescue analgesic consumption, shorter time to discharge from recovery room and from surgical ward, and the same incidence of side effects, comparably to controls.^7^

Our results, however, are at variance with the results of Konstantatos et al. They demonstrated that the addition of preprocedural oral oxycodone to morphine patient-controlled analgesia did not offer any analgesic advantage to patients undergoing uterine artery embolization.^13^ This could be ascribed to too late adminstration of oral oxycodone which was administered just before the procedure. It was proposed that adequate time interval between drug administration and surgical procedure was necessary for preemptive effect of oxycodone to be exhibited. The onset time of intravenous oxycodone is 2~3 minutes, and the peak time is 5 minutes, so 10 minutes before skin incision was chosen as the time of preemptive analgesia in our study.^11^,^12^,^14^,^15^

Opioids have several side effects such as postoperative nausea and vomiting (PONV), drowsiness, respiratory depression, gastrointestinal adverse effects and bladder dysfunction. PONV is one of the most undesirable postoperative outcomes for the patients. Furthermore, PONV leads to a delay in oral intake and duration recovery. The incidences of PONV, dizziness, itching were similar in both groups. No respiratory depression was observed in this study. The result was consistent with the report that oxycodone had less side effect profile than morphine.^10^-^12^

One limitation of our study is that we did not follow up the patients to evaluate whether chronic pain was reduced with the oxycodone used in the study. Future research should also focus on the long-term effects of oxycodone in reducing the incidence of chronic pain syndromes.

We conclude that preemptive oxycodone has a definitive role in decreasing hemodynamic changes, reducing postoperative pain and analgesic requirement in patients undergoing laparoscopic resection of ovarian tumor.
